# A Case of Leber's Hereditary Optic Neuropathy With Reversible Symmetric Lesions in the Substantia Nigra

**DOI:** 10.7759/cureus.76883

**Published:** 2025-01-03

**Authors:** Yasuyuki Takai, Akiko Yamagami, Mayumi Iwasa, Kenji Inoue, Ryoma Yasumoto, Hitoshi Ishikawa, Masato Wakakura

**Affiliations:** 1 Ophthalmology, Inouye Eye Hospital, Tokyo, JPN; 2 Opthalmology, Inouye Eye Hospital, Tokyo, JPN; 3 Clinical Laboratory, Kitazato University Hospital, Kanagawa, JPN; 4 Ophtalmology, Kitasato Universiity, Saganihara, JPN

**Keywords:** leber’s hereditary optic neuropathy, m.11778g>a variant, mitochondrial disease, substantia nigra, wernicke encephalopathy

## Abstract

A 34-year-old man with a history of alcoholism experienced progressive vision loss in both eyes over two months. His best corrected visual acuity was 0.1 OD and 0.2 OS, with visual field tests showing central scotoma bilaterally. Fundus examination revealed reddish optic discs with peripapillary telangiectasia in both eyes. Brain MRI showed bilateral high-intensity lesions in the substantia nigra on T2-weighted/Fluid-Attenuated Inversion Recovery (FLAIR) and diffusion-weighted images. Mitochondrial genetic analysis confirmed the m.11778G>A variant. After the patient stopped consuming alcohol and improved his nutrition, the substantia nigra lesions resolved 18 months after initial symptoms. The improvement of lesions following alcohol abstinence implies a possible link between nutritional status and substantia nigra abnormalities, suggesting concurrent alcohol encephalopathy. While substantia nigra lesions can complicate the diagnosis of Leber's hereditary optic neuropathy (LHON), careful assessment of alcohol consumption history and improvement following abstinence is essential for differential diagnosis.

## Introduction

Leber's hereditary optic neuropathy (LHON) is a mitochondrial disease that causes severe bilateral visual loss, typically in young men [1]. It is caused by genetic abnormalities in mitochondrial DNA, with the m.11778G>A, m.14484T>C, and m.3460G>A variants accounting for nearly 90% of cases. The m.11778G>A variant is the most common mitochondrial gene variant in LHON, impairing the complex I of the mitochondrial respiratory chain. While LHON typically affects only the optic nerve, rare cases present with other neurological abnormalities, such as dystonia and myoclonus, termed LHON plus. Although LHON typically shows normal brain MRI results, specific cases, particularly Harding disease, can exhibit intracranial demyelinating lesions [2]. Distinguishing LHON from other conditions can be challenging when intracranial abnormalities are detected. Here, we present a case of LHON with the m.11778G>A variant characterized by symmetric substantia nigra lesions in the midbrain, which were associated with comorbid alcohol encephalopathy.

## Case presentation

**Figure 1:**
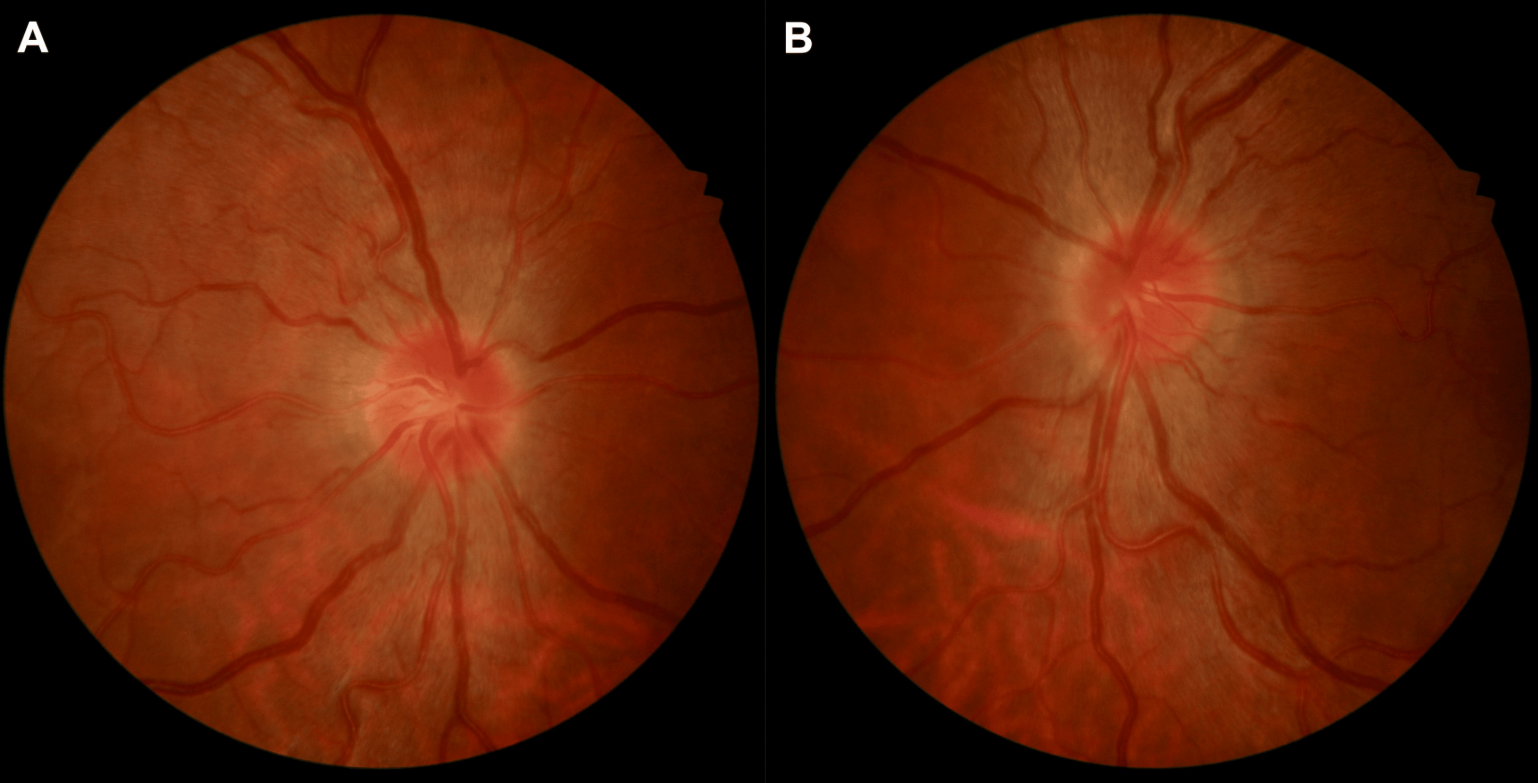
Fundus findings at the initial presentation. (A) and (B) In both eyes, redness and swelling of the optic disc and peripapillary telangiectasia were observed.

**Figure 2:**
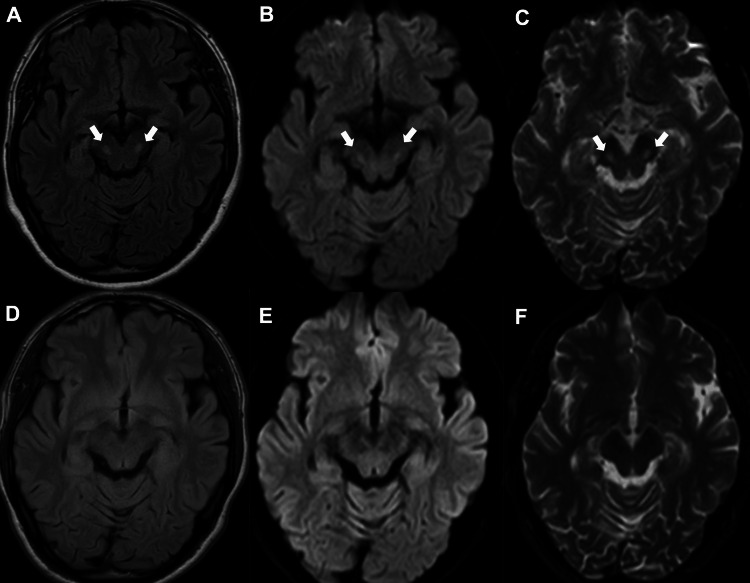
Brain MRI findings at the initial presentation and at 1.5 years after onset. At the initial presentation, T2-weighted/Fluid-Attenuated Inversion Recovery (FLAIR) images showed bilateral symmetric high signal intensity in the substantia nigra of the midbrain (A: arrows). (B, arrows) The diffusion-weighted images (DWIs) and (C, arrows) the apparent diffusion coefficient (ADC) map also showed symmetric high signal intensity in the same region. There were no abnormal findings in other areas, such as the mammillary bodies. At 1.5 years after onset, the high signal intensity in the substantia nigra of the midbrain had disappeared on (D) T2-weighted/FLAIR images, (E) DWI, and (F) ADC map.

A 34-year-old man with alcoholism and irregular dietary patterns presented with progressive bilateral visual disturbance for two months. Although his mother suffered from a gait disturbance and had received a diagnosis of progressive supranuclear palsy, she had no history of optic neuropathy. At the initial examination, his best-corrected visual acuity (BCVA) was reduced to 0.1 in the right eye and 0.2 in the left eye. The relative afferent pupillary defect was negative in both eyes. Goldman perimetry test revealed central scotomas in both eyes. Fundus examination revealed a reddish optic disc with peripapillary telangiectasia in both eyes (Figures 1A-1B). No other neurological abnormalities were observed. The routine blood test, which included a complete blood count (CBC) with hemoglobin levels and a basic metabolic panel, was normal. Vitamin B12 was 214 pg/mL (normal range: 180-914 pg/mL), and vitamin B1 was 33 ng/mL (normal range: 24-66 ng/mL), within the normal range. Although brain MRI showed no abnormalities in the optic nerves, symmetric high-intensity lesions were observed in the substantia nigra of the midbrain on T2-weighted/FLAIR, diffusion-weighted images (DWIs), and apparent diffusion coefficient (ADC) map (Figures 2A-2C). No abnormalities were found in other regions, including the mammillary bodies. A mitochondrial gene test was performed to differentiate subacute progressive optic neuropathy. The m.11778G>A variant was identified, leading to a diagnosis of LHON.

At seven months after onset, the BCVA had declined to 0.04 in both eyes. He attended psychiatric outpatient visits and received alcoholism treatment. After these treatments, the patient completely abstained from alcohol consumption and was able to maintain a regular dietary intake. At one year and six months after the onset, BCVA was slightly improved to 0.09 in the right eye and 0.07 in the left eye. The brain MRI revealed that the high-signal-intensity lesions on FLAIR/T2-weighted images, DWI, and ADC map in the substantia nigra of the midbrain had disappeared (Figures 2D-2F).

## Discussion

In this LHON case, in addition to the optic neuropathy, well-defined bilateral symmetric lesions were also observed in the substantia nigra of the midbrain. The bilateral substantia nigra lesions, which are characterized by their symmetry and well-defined borders, were more suggestive of systemic or metabolic disorders than inflammatory conditions, such as demyelinating diseases [3]. To date, including the present case, three comparable cases have been described in the literature (Table 1) [3-4]. All three cases were young men and presented only with the typical optic neuropathy that is seen in LHON with the m.11778G>A variant. The histories of severe alcohol consumption, such as alcohol dependence or alcoholic hepatitis, were noted. The MRI lesions were consistently localized to the substantia nigra of the midbrain. In the present case, due to a longer follow-up period compared to the other two cases, it was confirmed that the midbrain substantia nigra lesions disappeared in conjunction with alcohol abstinence, suggesting that these midbrain lesions were likely attributable to concurrent alcohol encephalopathy, such as Wernicke encephalopathy.

**Table 1:** Cases with the m.11778G>A mutation presenting with substantia nigra lesions in the midbrain. FLAIR, Fluid-Attenuated Inversion Recovery; T2WI, T2-weighted imaging; MRI, magnetic resonance imaging

Case	Age (years)/Gender	Other symptoms	Medical history	Family history	MRI lesions
Present case	34/male	None	Alcoholism	Mother: progressive supranuclear palsy	Bilateral symmetric T2WI/FLAIR high-intensity lesions in the substantia nigra Bilateral symmetric diffusion-weighted high-signal-intensity lesions in the substantia nigra No contrast-enhanced images were obtained No lesions in other regions
Chen et al. [3]	27/male	None	Alcoholic hepatitis, pancreatitis, and diabetes	None	Bilateral symmetric T2WI/FLAIR high-intensity lesions in the substantia nigra No diffusion-weighted images were obtained No contrast-enhanced lesions No lesions in other regions
Echiverri et al. [4]	29/male	None	Alcoholism	Maternal uncle: optic neuropathy	Bilateral symmetric T2WI/FLAIR high-intensity lesions in the substantia nigra No diffusion-weighted lesions No contrast-enhanced images were obtained No lesions in other regions

The substantia nigra lesions may be associated with Wernicke encephalopathy caused by thiamine deficiency due to alcohol abuse. MRI findings in Wernicke's encephalopathy typically show lesions in the mammillary bodies and periaqueductal area of the midbrain but rarely demonstrate lesions in the substantia nigra [5]. It has been reported that MRI abnormalities are reversible with treatment. Thiamine is involved in the TCA cycle within the mitochondria and plays a role in ATP production [6]. Even if blood thiamine levels are within the normal range, Wernicke's encephalopathy cannot be ruled out, and chronic alcohol intake can lead to dysfunction of thiamine-dependent enzymes [5]. Although thiamine deficiency was not evident in laboratory tests, malnutrition due to chronic alcoholism was present in this case. The resolution of midbrain lesions after alcohol abstinence suggests the possibility of comorbid alcohol encephalopathy.

The absence of typical Wernicke's encephalopathy lesions, such as in the mammillary bodies, and the presence of lesions confined to the substantia nigra might suggest the involvement of mitochondrial dysfunction. The absence of symptoms such as ophthalmoplegia and ataxia was also atypical for Wernicke encephalopathy. Substantia nigra lesions are commonly associated with Leigh's encephalopathy, and lesions in the substantia nigra have additionally been reported to occur with complex Ⅰ deficiency [7]. It was hypothesized that mitochondrial abnormalities might have conferred susceptibility to the pathology localized to the substantia nigra, which is among the rare MRI lesions observed in alcohol encephalopathy.

## Conclusions

This LHON patient with the m.11778G>A mutation presented with reversible symmetric lesions in the substantia nigra, complicated by concurrent alcohol encephalopathy. The fact that the lesion was limited to the substantia nigra suggests the possibility of mitochondrial disease involvement. While lesions in the substantia nigra can complicate the diagnosis of LHON, a careful assessment of alcohol consumption history and observation of improvement following abstinence are essential for an accurate differential diagnosis.
